# Coronary Microvascular Dysfunction in ANOCA and INOCA: From Pathophysiological Endotypes to Precision Diagnosis and Treatment

**DOI:** 10.3390/pathophysiology33040073

**Published:** 2026-09-28

**Authors:** Aladin Altic, Nikola Jovanovic, Haris Džinić, Ashwin Ragupathi, Franco Joaquin Cossettini, Anida Šehanović, Marko Baralic, Aleksandar Sič

**Affiliations:** 1 Department of Internal Medicine, Albert Einstein College of Medicine, Jacobi Medical Center/North Central Bronx Hospital, New York, NY 10467, USA; 2 Department of Internal Medicine, Cantonal Hospital “Dr. Irfan Ljubijankić”, 77000 Bihac, Bosnia and Herzegovina; 3 Department for Cardiovascular Surgery, University Clinical Center Sarajevo, 71000 Sarajevo, Bosnia and Herzegovina; 4 Nephrology Clinic, University Clinical Center of Serbia, 11000 Belgrade, Serbia; 5 Faculty of Medicine, University of Belgrade, 11000 Belgrade, Serbia

**Keywords:** precision medicine, coronary flow reserve, coronary vasomotor disorders, coronary microvascular dysfunction

## Abstract

Coronary microvascular dysfunction (CMD) is an important cause of angina and myocardial ischemia in patients without obstructive coronary artery disease and contributes substantially to the clinical spectrum of angina with non-obstructive coronary arteries (ANOCA) and ischemia with non-obstructive coronary arteries (INOCA). These conditions represent a heterogeneous group of coronary vasomotor disorders in which structural and functional abnormalities of the coronary microcirculation, endothelial dysfunction, coronary vasospasm, and diffuse non-obstructive atherosclerosis may occur alone or in combination. This review summarizes current knowledge on the pathophysiology, diagnosis, and management of CMD in patients with ANOCA and INOCA. We discuss the distinction between ANOCA and INOCA, the principal coronary functional endotypes, and the mechanisms responsible for impaired coronary microvascular function. We also review contemporary diagnostic approaches, including non-invasive imaging and invasive coronary function testing, with emphasis on their complementary roles in identifying the underlying mechanism of ischemia. Current management strategies are presented from a mechanism-based perspective, including lifestyle modification, pharmacological therapy, endotype-guided treatment, and emerging therapeutic approaches. Improved understanding of coronary functional abnormalities has shifted clinical evaluation beyond the exclusion of obstructive coronary artery disease toward identification of the mechanism responsible for persistent symptoms and ischemia. Continued advances in disease phenotyping, coronary function testing, and targeted therapies are expected to support more individualized management and improve outcomes in this diverse patient population.

## 1. Introduction

Coronary artery disease (CAD) is the leading cause of morbidity and mortality worldwide, with chronic coronary syndromes representing one of the most common reasons for cardiovascular evaluation [1,2]. For decades, myocardial ischemia was primarily attributed to flow-limiting stenoses of the epicardial coronary arteries and diagnostic strategies were largely directed toward identifying anatomically significant obstructive disease [3,4]. Although this concept remains fundamental, it does not explain why many patients present with angina or objective evidence of myocardial ischemia despite the absence of obstructive coronary artery disease on coronary angiography. It is now well recognized that abnormalities affecting both the epicardial coronary arteries and the coronary microcirculation may impair myocardial perfusion and produce ischemic symptoms [5,6].

Among patients referred for invasive coronary angiography because of suspected chronic coronary syndromes, non-obstructive coronary arteries are identified more frequently than previously appreciated. This clinical scenario is particularly common in women but also affects a substantial proportion of men [7,8]. Despite the absence of obstructive coronary lesions, many of these patients continue to experience recurrent angina, reduced exercise capacity, impaired quality of life, repeated hospital admissions and multiple diagnostic procedures [7,9]. These symptoms were often attributed to non-cardiac causes or considered reassuring after a “normal” coronary angiogram. However, these patients remain at increased cardiovascular risk and frequently remain undertreated because the underlying mechanism of ischemia is not identified [10,11].

Coronary microvascular dysfunction (CMD) is one of the principal mechanisms responsible for myocardial ischemia in patients without obstructive epicardial coronary artery disease [12]. Structural remodeling of the coronary microcirculation, impaired vasodilatory capacity, endothelial dysfunction and abnormal vasoconstriction may all reduce myocardial blood flow despite angiographically normal or mildly diseased epicardial coronary arteries [12,13]. In many patients, CMD coexists with coronary vasospasm or diffuse non-obstructive atherosclerosis, indicating that myocardial ischemia often results from several interacting mechanisms rather than a single pathological process [14,15,16].

This review summarizes current knowledge on coronary microvascular dysfunction in patients with angina or ischemia and non-obstructive coronary arteries. We first define the concepts of ANOCA and INOCA, then discuss coronary functional endotypes, followed by the pathophysiological mechanisms of coronary microvascular dysfunction, contemporary diagnostic approaches, current treatment strategies, and future directions. By bringing these concepts together, this review aims to provide clinicians with a practical overview of the disease and support a better understanding of its pathophysiology, diagnosis and management.

## 2. ANOCA and INOCA: Definitions and Clinical Endotypes

### 2.1. ANOCA Versus INOCA

The terms angina with non-obstructive coronary arteries (ANOCA) and ischemia with non-obstructive coronary arteries (INOCA) were introduced to describe patients with anginal symptoms or objective evidence of myocardial ischemia despite the absence of obstructive epicardial coronary artery disease. Although these terms are frequently used together, they do not describe identical clinical populations [17,18].

ANOCA refers to patients with anginal symptoms or anginal equivalents in the absence of hemodynamically significant epicardial coronary artery stenosis. The diagnosis is primarily based on the clinical presentation after obstructive coronary artery disease has been excluded by invasive coronary angiography or coronary computed tomography angiography, with physiological assessment of intermediate lesions using fractional flow reserve (FFR) or instantaneous wave-free ratio (iFR) when appropriate. Objective evidence of myocardial ischemia is not required for the diagnosis of ANOCA, as conventional stress testing may be normal or inconclusive despite persistent symptoms [19,20].

INOCA is a more specific clinical entity in which patients have both non-obstructive coronary arteries and objective evidence of myocardial ischemia demonstrated by non-invasive or invasive functional testing. Ischemia may be documented using stress echocardiography, positron emission tomography, cardiovascular magnetic resonance, single-photon emission computed tomography, or invasive coronary function testing. INOCA can also be considered a subgroup within the broader ANOCA population in whom ischemia has been objectively confirmed [11,21].

Although these terms are sometimes used together, the distinction is clinically relevant. ANOCA is defined by symptoms, whereas INOCA additionally requires objective documentation of myocardial ischemia. This distinction influences the diagnostic pathway, interpretation of test results and subsequent clinical management [11,21].

### 2.2. Coronary Functional Endotypes

Although ANOCA and INOCA describe patients according to symptoms and the presence or absence of documented myocardial ischemia, they do not identify the mechanism responsible for myocardial ischemia. Patients with these syndromes are a heterogeneous population in whom similar clinical presentations may result from different coronary vasomotor abnormalities. For this reason, diagnostic strategies extend beyond the identification of non-obstructive coronary arteries and aim to determine the underlying coronary functional abnormality [7,19,22].

The principal coronary functional endotypes include endothelial dysfunction, structural coronary microvascular dysfunction, functional coronary microvascular dysfunction, epicardial vasospastic angina, microvascular vasospastic angina and mixed endotypes in which more than one mechanism is present simultaneously. These abnormalities differ in their underlying biology, clinical presentation, diagnostic findings, prognosis, and response to treatment. Individual patients frequently exhibit more than one functional abnormality, showing the complexity of coronary vasomotor disease [23,24].

Identification of the underlying coronary functional endotype has important clinical implications because it provides a mechanistic explanation for persistent symptoms and supports a more individualized diagnostic and therapeutic approach [25,26]. The principal coronary functional endotypes and their diagnostic characteristics are summarized in Table 1. The mechanisms responsible for each endotype are discussed in the following section.

## 3. Pathophysiology of Coronary Microvascular Dysfunction

### 3.1. Structural Coronary Microvascular Dysfunction

Structural CMD is characterized by permanent anatomical changes within the coronary microcirculation that limit its ability to increase blood flow during periods of increased myocardial oxygen demand. Structural CMD develops as a consequence of remodeling of the microvascular network itself. These changes reduce the capacity of the coronary circulation to augment myocardial perfusion, resulting in impaired hyperemic blood flow and increased microvascular resistance [13,29,30].

Microvascular remodeling primarily affects the intramyocardial arterioles and capillaries, the vessels responsible for regulating coronary vascular resistance and matching myocardial blood flow to local metabolic demand [31,32]. One of the main features of structural CMD is inward remodeling of the arterioles, characterized by an increased wall-to-lumen ratio that narrows the vascular lumen without necessarily changing the external vessel diameter. This process increases resistance to blood flow and limits the maximal vasodilatory capacity of the microcirculation. At the same time, capillary rarefaction, defined as a reduction in myocardial capillary density, decreases the total cross-sectional area available for tissue perfusion, further restricting oxygen delivery during stress. Perivascular fibrosis and extracellular matrix deposition may further stiffen the microvascular wall and impair its ability to respond appropriately to changes in myocardial oxygen demand [33,34,35].

These structural abnormalities usually develop gradually as a consequence of chronic exposure to cardiovascular risk factors rather than as isolated pathological processes. Hypertension promotes inward remodeling of resistance vessels through smooth muscle hypertrophy and increased collagen deposition, while diabetes mellitus accelerates microvascular remodeling through chronic inflammation, oxidative stress, advanced glycation end products, and endothelial injury [36,37,38]. Similar changes have been described in chronic kidney disease, obesity, aging, and diffuse atherosclerosis, where persistent vascular injury progressively alters the architecture of the coronary microcirculation [13,39,40]. Structural CMD is also commonly observed in conditions associated with left ventricular hypertrophy, including hypertensive heart disease and hypertrophic cardiomyopathy, in which myocardial growth is not accompanied by a proportional expansion of the coronary microvascular network [32,41].

The physiological consequence of this remodeling is a reduction in coronary flow reserve caused by an inability of the microcirculation to adequately increase blood flow during hyperemia. Because resting myocardial perfusion is often preserved, patients may remain asymptomatic at rest, while ischemia develops when myocardial oxygen demand increases during physical or emotional stress. Invasive coronary physiology typically demonstrates reduced coronary flow reserve together with increased minimal microvascular resistance, reflecting the fixed structural limitation of the coronary microcirculation rather than a transient vasomotor disturbance [42,43].

Structural and functional CMD are described as distinct endotypes, they frequently overlap in clinical practice. Structural remodeling is commonly accompanied by endothelial dysfunction and impaired vasodilatory signaling, which makes it difficult to separate fixed anatomical abnormalities from superimposed functional disturbances. Distinguishing structural CMD is clinically relevant because it reflects chronic remodeling of the coronary microcirculation and is often associated with longstanding cardiovascular risk factors and myocardial remodeling [16,44].

### 3.2. Functional Coronary Microvascular Dysfunction

Functional coronary microvascular dysfunction reflects abnormal regulation of coronary vasomotor tone rather than permanent remodeling of the microcirculation. In these patients, resistance vessels retain their structural integrity but respond inappropriately to physiological or pharmacological stimuli, resulting in an inadequate increase in myocardial perfusion or, in some cases, paradoxical vasoconstriction. This dynamic nature distinguishes functional CMD from the fixed flow limitation produced by structural remodeling [42,45].

The regulation of coronary vascular tone depends on a complex interaction between endothelial cells, vascular smooth muscle cells, autonomic neural input, and locally released vasoactive substances. Dysfunction at any of these levels may disturb the balance between vasodilation and vasoconstriction. Rather than representing a single abnormality, functional CMD is better viewed as a disorder of coronary vasomotor control in which several regulatory pathways may be impaired simultaneously [16,46,47].

Endothelial dysfunction is the best-characterized mechanism within this endotype. Reduced nitric oxide availability limits endothelium-dependent vasodilation, while oxidative stress accelerates nitric oxide degradation and promotes a pro-inflammatory vascular environment. At the same time, increased production of vasoconstrictors such as endothelin-1 favors persistent vasoconstrictor tone. These abnormalities reduce coronary vasodilatory capacity even in the absence of visible structural changes within the microcirculation [48,49].

Functional impairment may also originate beyond the endothelium. Abnormal relaxation of vascular smooth muscle cells has been demonstrated by attenuated responses to endothelium-independent vasodilators, suggesting that the ability of resistance vessels to relax may be intrinsically impaired. Enhanced sympathetic activation further increases microvascular tone and may contribute to exercise-induced or stress-induced ischemia in susceptible individuals [50,51].

These abnormalities are commonly associated with hypertension, diabetes mellitus, dyslipidemia, obesity, smoking, chronic kidney disease, and chronic inflammatory conditions. Because functional disturbances often develop before permanent remodeling becomes established, they may represent an early stage of coronary microvascular disease and provide an opportunity for intervention through intensive risk factor modification and targeted pharmacological therapy [52].

### 3.3. Endothelial Dysfunction, Inflammation and Oxidative Stress

Endothelial dysfunction is a central mechanism linking structural and functional abnormalities of the coronary microcirculation. While structural remodeling limits the maximal capacity of the microvascular network and functional CMD reflects impaired vasomotor regulation, endothelial dysfunction disrupts the biological processes that normally maintain vascular homeostasis. Healthy endothelial cells continuously adjust coronary blood flow by releasing vasodilators, inhibiting inflammation and thrombosis, and preserving the balance between vascular relaxation and constriction. When endothelial function becomes impaired, these protective mechanisms gradually deteriorate, promoting myocardial ischemia even in the absence of obstructive epicardial coronary disease [19,53,54].

Reduced NO bioavailability is the hallmark of endothelial dysfunction. Under physiological conditions, endothelial NO synthase generates NO, which diffuses into vascular smooth muscle cells and induces vasodilation while also suppressing platelet activation, leukocyte adhesion, and vascular inflammation. In CMD, oxidative stress decreases NO availability through several complementary mechanisms. Reactive oxygen species rapidly inactivate NO by forming peroxynitrite, while oxidative injury also uncouples endothelial NO synthase, reducing NO production and further increasing reactive oxygen species generation. This self-perpetuating cycle progressively shifts the coronary microcirculation toward vasoconstriction rather than vasodilation [55,56,57]. Because NO bioavailability reflects the balance between its endothelial production and oxidative degradation, this pathway also has therapeutic implications. Statins may improve endothelial function not only through lipid lowering but also by reducing oxidative stress and enhancing endothelial NO synthase activity. Although antioxidants could theoretically preserve NO bioavailability, current clinical evidence does not support their routine use specifically for CMD [55,56,57].

Inflammation further amplifies endothelial dysfunction. Chronic exposure to cardiovascular risk factors, including hypertension, diabetes mellitus, obesity, chronic kidney disease, and smoking, promotes persistent low-grade vascular inflammation. Pro-inflammatory cytokines such as tumor necrosis factor-α and interleukin-6 impair endothelial signaling, reduce NO synthesis, and increase the expression of adhesion molecules that facilitate leukocyte recruitment into the vascular wall. These inflammatory changes not only worsen endothelial function but also contribute to progressive microvascular injury over time [58,59]. At the same time, endothelial dysfunction favors the production of potent vasoconstrictors, particularly endothelin-1. Increased endothelin-1 activity enhances basal coronary vascular tone, opposes NO-mediated relaxation, and increases the susceptibility of both epicardial and microvascular vessels to vasospasm. Activation of downstream signaling pathways, including RhoA/Rho-kinase, further augments smooth muscle contraction and contributes to persistent abnormalities in coronary vasomotor control [60,61].

Endothelial dysfunction, inflammation, and oxidative stress reinforce one another throughout the course of CMD. Endothelial injury promotes oxidative stress, oxidative stress intensifies inflammation, and persistent inflammation further impairs endothelial function. This interaction helps explain why CMD frequently progresses despite the absence of significant epicardial stenosis and highlights the importance of therapies that target vascular health in addition to symptom relief [62,63].

### 3.4. Coronary Microvascular Spasm

Coronary microvascular spasm is a functional abnormality characterized by transient constriction of the coronary microcirculation that reduces myocardial blood flow despite the absence of significant epicardial coronary artery narrowing [64,65]. Microvascular spasm reflects an exaggerated vasoconstrictor response that is typically transient and reversible. During these episodes, myocardial oxygen supply becomes insufficient to meet metabolic demand, resulting in transient ischemia and anginal symptoms [66].

Microvascular spasm may occur spontaneously or be triggered by physical exertion, emotional stress, cold exposure, or pharmacological stimuli. Because the affected vessels are too small to be visualized by conventional coronary angiography, diagnosis relies on functional rather than anatomical assessment. During acetylcholine provocation testing, patients typically develop reproduction of their usual angina together with ischemic electrocardiographic changes, but without significant epicardial coronary artery constriction. This pattern distinguishes microvascular spasm from epicardial vasospastic angina while confirming that ischemia originates within the coronary microcirculation [67,68].

Microvascular spasm frequently coexists with other coronary vasomotor abnormalities. Many patients demonstrate overlapping features of endothelial dysfunction, impaired coronary flow reserve, or structural CMD, which indicates that myocardial ischemia often reflects the interaction of several mechanisms rather than a single isolated abnormality [69].

### 3.5. Interaction with Diffuse Atherosclerosis

Coronary microvascular dysfunction and diffuse non-obstructive atherosclerosis frequently coexist and should not be viewed as independent disease processes. Although patients with ANOCA or INOCA lack flow-limiting epicardial stenoses, many have diffuse atherosclerotic plaque throughout the coronary tree. This pattern of disease often remains angiographically subtle but may still impair myocardial perfusion and contribute to persistent ischemic symptoms. CMD develops in the setting of diffuse atherosclerosis far more often than in completely normal coronary arteries [70,71].

Diffuse atherosclerosis influences myocardial perfusion through several complementary mechanisms. Progressive plaque accumulation causes mild luminal narrowing along long segments of the epicardial coronary arteries, producing cumulative resistance to coronary blood flow even when no individual lesion meets the threshold for obstructive disease. At the same time, chronic endothelial injury, vascular inflammation and oxidative stress extend into the coronary microcirculation, promoting endothelial dysfunction, impaired vasodilatory capacity, and structural remodeling of resistance vessels. As a result, abnormalities within the epicardial arteries and the microcirculation often evolve in parallel rather than independently [72,73,74].

This interaction also helps explain why symptoms and objective ischemia may appear disproportionate to angiographic findings. Mild diffuse epicardial disease may modestly reduce coronary pressure, while coexisting CMD limits the ability of the microcirculation to compensate by increasing myocardial blood flow during stress. The combined effect is a reduction in coronary flow reserve that cannot be attributed to either abnormality alone. Patients with diffuse non-obstructive atherosclerosis may continue to experience angina despite the absence of focal obstructive lesions on coronary angiography [75,76].

Diffuse non-obstructive atherosclerosis and CMD often contribute to myocardial ischemia simultaneously, even when coronary angiography shows no flow-limiting stenosis. Evaluating both the epicardial coronary arteries and the coronary microcirculation therefore provides a more complete explanation for persistent symptoms and objective evidence of ischemia in patients with ANOCA and INOCA [77,78]. The principal mechanisms involved in coronary microvascular dysfunction and their characteristic features are summarized in Table 2.

## 4. Clinical Presentation and Diagnostic Evaluation

### 4.1. Clinical Manifestations

Patients with coronary microvascular dysfunction usually present with symptoms of myocardial ischemia despite the absence of obstructive epicardial coronary artery disease. Most report chest pain, but many also experience exertional dyspnea, reduced exercise tolerance, fatigue, or palpitations. Because these symptoms closely resemble those of obstructive coronary artery disease, clinicians cannot distinguish the two conditions on clinical presentation alone. Many patients therefore undergo repeated diagnostic evaluations before clinicians identify the underlying coronary vasomotor disorder [79,80,81].

Chest pain most often develops during physical exertion or emotional stress, but many patients also report symptoms at rest. Patients usually describe the discomfort as pressure, tightness, heaviness, or burning in the retrosternal region. Some experience prolonged episodes that continue after exercise, whereas others develop intermittent symptoms without a consistent trigger. Dyspnea may accompany chest pain or replace it completely as an ischemic equivalent. Women more frequently report atypical symptoms, including fatigue, nausea, or pain radiating to the jaw, neck, or upper extremities [82,83].

The underlying coronary endotype also influences symptom patterns. Structural and functional CMD usually produce exertional angina because the coronary microcirculation cannot adequately increase myocardial blood flow during periods of increased oxygen demand. Coronary vasospasm more often causes symptoms at rest, particularly during the night or early morning [84,85].

Symptoms often persist for months or years and substantially affect daily life. Recurrent chest pain limits physical activity, reduces quality of life, and frequently leads to repeated emergency department visits, hospital admissions, and additional cardiac testing. Many patients become frustrated when coronary angiography excludes obstructive disease but their symptoms continue without a clear explanation [86,87].

Clinical presentation alone cannot identify the underlying mechanism of ischemia. Sex differences are important in ANOCA and INOCA. Women, particularly after menopause, more commonly develop endothelial and microvascular dysfunction, possibly because of hormonal changes, reduced nitric oxide bioavailability, inflammation, and autonomic dysfunction. They also more often report dyspnea, fatigue, prolonged chest pain, and symptoms at rest. Although treatment should primarily follow the identified endotype, hormonal status, cardiovascular risk factors, and drug tolerance should also be considered [8,18,82,83]. Physicians therefore need functional assessment of the coronary circulation to distinguish CMD from other causes of chest pain and to identify the specific coronary endotype that guides further management [49,88].

### 4.2. Non-Invasive Assessment

Non-invasive testing is crucial in the evaluation of patients with suspected coronary microvascular dysfunction [18]. The diagnostic workup begins by excluding obstructive epicardial coronary artery disease and identifying objective evidence of myocardial ischemia. Standard stress testing may detect ischemia but cannot determine whether symptoms originate from the epicardial coronary arteries or the coronary microcirculation. For this reason, contemporary imaging techniques increasingly focus on quantifying myocardial blood flow and coronary flow reserve rather than simply detecting perfusion defects [19,89,90].

Coronary computed tomography angiography (CCTA) provides an excellent anatomical assessment of the epicardial coronary arteries and represents the preferred non-invasive method for excluding obstructive coronary artery disease in many patients with stable chest pain [91]. Although CCTA does not directly diagnose CMD, it identifies diffuse non-obstructive atherosclerosis, excludes significant epicardial stenosis, and helps select patients who require further functional evaluation. Combining anatomical and functional imaging also improves the diagnostic evaluation of patients with ANOCA and INOCA [7,17].

Positron emission tomography (PET) provides the most validated non-invasive assessment of coronary microvascular function [17,90]. PET quantifies absolute myocardial blood flow at rest and during pharmacologically induced hyperemia, allowing calculation of coronary flow reserve (CFR). Reduced CFR in the absence of obstructive coronary artery disease strongly supports the diagnosis of CMD and carries important prognostic value. High reproducibility and extensive clinical validation have established PET as the non-invasive reference standard for evaluating coronary microvascular function, although limited availability, radiation exposure, and cost restrict its widespread use [92,93].

Stress cardiovascular magnetic resonance (CMR) has become an important alternative to PET. Quantitative perfusion imaging measures myocardial blood flow and myocardial perfusion reserve without exposing patients to ionizing radiation while simultaneously providing detailed information about ventricular structure, function, and myocardial tissue characteristics. The high spatial resolution of CMR also improves the detection of diffuse subendocardial ischemia, which often escapes conventional perfusion imaging. Scanner availability, longer examination times, and technical expertise currently limit its broader implementation [94,95].

Stress echocardiography primarily detects regional wall motion abnormalities caused by obstructive coronary artery disease and has limited sensitivity for CMD because microvascular ischemia often produces diffuse subendocardial hypoperfusion rather than focal contractile abnormalities [96]. Doppler assessment of coronary flow velocity reserve in the left anterior descending artery improves the diagnostic value of echocardiography by providing a functional estimate of coronary flow reserve. This technique remains attractive because it is widely available, inexpensive, and free of radiation, but operator dependence and technical limitations reduce its reproducibility [97].

The reported diagnostic accuracy of non-invasive methods varies according to the imaging protocol, selected cut-off value, and invasive reference standard. In the WISE study, a CMR myocardial perfusion reserve index threshold of 1.84 identified abnormal invasive coronary reactivity testing with 73% sensitivity and 74% specificity. A systematic review of 37 studies found that CFR below 2.0 was the most commonly used threshold for PET and transthoracic Doppler echocardiography. However, substantial methodological heterogeneity prevented the calculation of reliable pooled sensitivity and specificity values. Furthermore, these methods primarily identify impaired flow reserve and cannot reliably distinguish structural from functional CMD or directly diagnose endothelial dysfunction and vasospasm [98,99].

No single non-invasive modality identifies every coronary functional abnormality. PET and stress CMR primarily evaluate impaired coronary flow reserve, whereas endothelial dysfunction and coronary vasospasm usually require invasive coronary function testing [27,28]. Clinicians should therefore select the diagnostic strategy according to the clinical presentation, local expertise, and the coronary mechanism most strongly suspected. The invasive assessment of coronary microvascular function is discussed in the following section.

### 4.3. Invasive Coronary Function Testing

Invasive coronary function testing (ICFT) provides the most comprehensive assessment of coronary microvascular function and now serves as the reference standard for identifying the mechanisms responsible for ANOCA and INOCA. Unlike conventional coronary angiography, which evaluates only the anatomy of the epicardial coronary arteries, ICFT directly assesses coronary vasodilator capacity, microvascular resistance, endothelial function, and vasospastic responses during a single procedure. This comprehensive approach allows clinicians to identify the underlying coronary endotype and tailor treatment accordingly [27,28,100].

The procedure usually begins after coronary angiography excludes flow-limiting epicardial stenosis. A pressure-temperature or Doppler guidewire is advanced into a major coronary artery to measure physiological indices at rest and during pharmacologically induced hyperemia. Adenosine produces maximal vasodilation and allows calculation of coronary flow reserve (CFR) and the index of microcirculatory resistance (IMR). CFR reflects the ability of the coronary circulation to increase blood flow during increased myocardial demand, whereas IMR provides a direct estimate of microvascular resistance that is less influenced by hemodynamic conditions. A reduced CFR and an elevated IMR support the diagnosis of structural coronary microvascular dysfunction [101,102]. In clinical practice, structural CMD is suggested by reduced CFR (<2.5) combined with increased minimal microvascular resistance, defined as IMR ≥ 25 or HMR > 2.5. This pattern indicates impaired hyperemic flow caused by fixed microvascular remodeling. In contrast, functional CMD is characterized by reduced CFR despite normal microvascular resistance, often because resting coronary flow is abnormally elevated while minimal resistance during hyperemia remains preserved. Assessment of resting and hyperemic flow, IMR or HMR, and microvascular resistance reserve can therefore help distinguish these endotypes in an individual patient. However, these are physiological classifications rather than direct anatomical measurements and overlap between endotypes remains common [18,101,102].

ICFT also evaluates endothelial function and coronary vasospasm. Incremental intracoronary acetylcholine administration assesses the vasomotor response of both the epicardial coronary arteries and the coronary microcirculation. Healthy vessels dilate in response to acetylcholine, whereas endothelial dysfunction produces paradoxical vasoconstriction. Higher acetylcholine doses may provoke epicardial or microvascular spasm, reproducing the patient’s symptoms together with ischemic electrocardiographic changes. This protocol distinguishes epicardial vasospastic angina from microvascular vasospasm and identifies patients with mixed coronary vasomotor disorders [103,104].

Some centers use ergonovine as an alternative provocative agent. Acetylcholine acts through muscarinic receptors and assesses endothelium-dependent vasomotion, whereas ergonovine induces vascular smooth muscle contraction mainly through serotonergic receptors. Acetylcholine more often provokes diffuse, distal, or microvascular spasm, while ergonovine tends to produce focal and proximal epicardial spasm. Therefore, the two agents provide complementary rather than identical diagnostic information [105].

The combination of adenosine- and acetylcholine-based testing allows clinicians to differentiate several functional endotypes, including structural CMD, functional CMD, endothelial dysfunction, epicardial vasospasm, microvascular vasospasm, and overlapping phenotypes. This information explains persistent symptoms more accurately than coronary angiography alone and provides the physiological basis for individualized therapy [26,104].

Recent ESC guidelines place greater emphasis on ICFT in patients with persistent angina despite non-obstructive coronary arteries. The procedure has demonstrated a favorable safety profile when performed in experienced centers and now represents an important component of precision medicine for patients with ANOCA and INOCA. Table 3 compares the principal non-invasive and invasive diagnostic modalities used to evaluate coronary microvascular dysfunction [21,27].

## 5. Management of Coronary Microvascular Dysfunction

### 5.1. Lifestyle and Risk Factor Modification

Lifestyle optimization and aggressive control of cardiovascular risk factors form the foundation of treatment for patients with coronary microvascular dysfunction [103]. Unlike obstructive coronary artery disease, where treatment often focuses on relieving flow-limiting stenoses, management of CMD aims to improve endothelial function, reduce microvascular dysfunction, slow vascular remodeling, and alleviate symptoms. Most recent guidelines recommend that every patient with ANOCA or INOCA receive individualized counseling on lifestyle modification regardless of the underlying coronary endotype [75,106].

Regular physical activity is one of the most effective non-pharmacological interventions. Aerobic exercise improves endothelial function, increases nitric oxide bioavailability, reduces oxidative stress, and enhances exercise capacity. Cardiac rehabilitation programs have also improved symptom burden, quality of life, and functional capacity in patients with ANOCA and CMD. Moderate-intensity aerobic exercise may be particularly beneficial in structural CMD and endothelial dysfunction by improving vascular function and cardiovascular risk factors. In patients with vasospastic or exercise-induced symptoms, supervised low-to-moderate-intensity aerobic training with gradual progression may be more appropriate, while resistance training can complement aerobic exercise in patients with reduced functional capacity. Exercise intensity should be individualized, particularly in patients with exercise-induced angina or vasospastic disease, where gradual progression and appropriate supervision may improve adherence while minimizing symptom provocation [107,108].

Weight management and dietary modification further support coronary microvascular health. A Mediterranean-style diet rich in fruits, vegetables, whole grains, legumes, fish, and unsaturated fats improves lipid profiles, reduces systemic inflammation, and promotes endothelial function. In overweight or obese patients, sustained weight loss has been associated with improvements in angina symptoms, exercise tolerance, and coronary microvascular function. These benefits appear to result from the combined effects of reduced inflammation, improved insulin sensitivity, and lower vascular oxidative stress [109,110].

Smoking cessation remains a priority because tobacco exposure directly impairs endothelial function, increases oxidative stress, and promotes coronary vasoconstriction. Smoking also increases the risk of coronary vasospasm and accelerates diffuse atherosclerosis, both of which frequently coexist with CMD. Blood pressure, lipid levels, glycemic control, and body weight should therefore be managed according to cardiovascular prevention guidelines, as these factors contribute directly to coronary microvascular remodeling and endothelial dysfunction [111].

Psychological health also influences symptom burden in patients with CMD. Chronic stress, anxiety, and depression increase sympathetic activation, worsen endothelial dysfunction, and reduce adherence to lifestyle recommendations and medical therapy. Stress reduction strategies, behavioral interventions, and participation in multidisciplinary rehabilitation programs may therefore complement conventional cardiovascular treatment by improving both symptoms and quality of life [112,113].

Lifestyle modification alone rarely eliminates symptoms in symptomatic patients, but it provides the foundation upon which pharmacological and endotype-guided therapies are built. Early and sustained control of cardiovascular risk factors may also slow disease progression and improve long-term cardiovascular outcomes.

### 5.2. Pharmacological Treatment

Drug therapy aims to relieve angina, improve coronary microvascular function, and treat the mechanisms responsible for myocardial ischemia. Because ANOCA and INOCA include several different coronary endotypes, patients do not respond equally to the same medications. Treatment should therefore be selected according to the underlying coronary functional abnormality whenever possible rather than using a uniform approach for all patients [114,115].

Patients with structural CMD usually receive medications that reduce myocardial oxygen demand and improve coronary perfusion. Beta-blockers are commonly used because they lower heart rate, prolong diastole, and increase myocardial perfusion time. Vasodilating beta-blockers, such as nebivolol, may provide additional benefits. In addition to selective β1-adrenergic receptor blockade, nebivolol promotes nitric oxide-mediated vasodilation, which may improve endothelial function and coronary blood flow. This mechanism may be particularly relevant in patients with CMD associated with endothelial dysfunction. Carvedilol blocks β1-, β2-, and α1-adrenergic receptors, reducing myocardial oxygen demand while promoting vasodilation. Its antioxidant effects may also improve nitric oxide bioavailability and endothelial function [116,117]. ACE inhibitors are also frequently prescribed because they improve endothelial function, increase nitric oxide bioavailability, and may improve coronary flow reserve. Statins complement these effects by reducing inflammation, improving endothelial function, and slowing the progression of diffuse atherosclerosis [72,118].

Calcium channel blockers are preferred in patients with epicardial or microvascular vasospasm because they reduce coronary vasoconstriction and improve myocardial perfusion. Patients with more severe vasospastic disease may require higher doses or a combination of different calcium channel blockers. Short-acting nitrates provide rapid symptom relief during acute episodes, whereas long-acting nitrates or nicorandil may be considered when symptoms persist. However, long-acting nitrates are often ineffective or poorly tolerated in microvascular angina and may worsen symptoms through a coronary steal effect. Continuous use may also lead to nitrate tolerance, while evidence from endotype-specific randomized trials remains limited [11]. In contrast, non-selective beta-blockers should generally be avoided in patients with predominant vasospastic disease because they may aggravate coronary vasoconstriction [19,84].

Several additional antianginal agents may be considered when symptoms remain uncontrolled. Ranolazine reduces intracellular sodium and calcium overload and may improve myocardial relaxation without significantly affecting heart rate or blood pressure. Patients with markedly reduced coronary flow reserve appear to benefit the most. Ivabradine may be useful in selected patients with elevated heart rate, while trimetazidine and nicorandil remain alternative options where available [51,118,119].

Many patients require a combination of medications together with lifestyle modification and intensive control of cardiovascular risk factors. Identifying the underlying coronary endotype remains the best strategy for selecting the most appropriate treatment, which is discussed in the following section.

### 5.3. Endotype-Guided Therapy

For many years, treatment was largely empirical because the mechanism responsible for ischemia remained unknown after obstructive coronary artery disease had been excluded. The introduction of invasive coronary function testing changed this approach by allowing clinicians to distinguish structural CMD, functional CMD, endothelial dysfunction, epicardial vasospasm, microvascular vasospasm, and mixed endotypes during a single procedure. This physiological classification provides a direct explanation for persistent symptoms and helps match treatment to the underlying mechanism rather than relying on symptom relief alone [21,23,87,118].

The clinical value of this strategy was evaluated in CorMicA, a single-center randomized controlled trial involving 151 patients with angina or evidence of ischemia and no obstructive coronary artery disease. All patients underwent invasive testing with CFR, IMR, FFR, and acetylcholine, after which they were randomized to endotype-guided treatment based on disclosed test results (n = 75) or standard care with blinded results (n = 76). The primary endpoint was the change in the Seattle Angina Questionnaire summary score at 6 months, while prespecified follow-up at 1 year assessed angina, quality of life, and major adverse cardiovascular events. At 1 year, the intervention group had a 13.6-point greater improvement in angina score and better quality of life. However, cardiovascular event rates were similar between the groups after a median follow-up of 19 months. CorMicA therefore provides proof-of-concept evidence that endotype-guided treatment improves symptoms and quality of life, but larger multicenter studies are needed to determine its effect on major cardiovascular outcomes [120,121].

Endotype-guided management also helps explain why some patients fail to respond to conventional therapy. Persistent symptoms do not necessarily indicate inadequate treatment but may reflect an incorrect diagnosis or the presence of overlapping mechanisms. For example, a patient with microvascular vasospasm is unlikely to improve with the same strategy used for predominant structural CMD, while individuals with mixed endotypes often require combinations of therapies targeting both impaired vasodilation and abnormal vasoconstriction. Defining the underlying mechanism therefore reduces trial-and-error prescribing and allows treatment to be adjusted more rationally [25,122,123].

This approach also has practical implications beyond drug selection. Identifying the coronary endotype provides patients with a clear explanation for persistent symptoms despite the absence of obstructive coronary artery disease, reduces unnecessary repeat investigations, and supports more informed long-term follow-up. It also facilitates communication between clinicians by replacing the broad diagnosis of ANOCA or INOCA with a specific physiological abnormality that can guide future management [15,84,124].

Coronary function testing requires specialized equipment, standardized protocols, and experienced operators, and it is still unavailable in many centers. Wider adoption of this technique will likely be necessary before endotype-guided management becomes routine clinical practice. So, despite these advantages, routine implementation is still limited.

### 5.4. Emerging Therapies

Current treatment of coronary microvascular dysfunction is largely based on medications originally developed for other cardiovascular conditions. Although these therapies improve symptoms in many patients, they do not specifically target the molecular mechanisms responsible for endothelial dysfunction, microvascular remodeling, or coronary vasomotor abnormalities. As a result, several novel therapies are now being evaluated with the goal of modifying the underlying disease process rather than simply relieving angina [125,126].

One of the most promising therapeutic targets is the endothelin pathway. Endothelin-1 is mainly produced by endothelial cells and regulates coronary vascular tone through endothelin A (ETA) and endothelin B (ETB) receptors. ETA receptors on vascular smooth muscle cells promote calcium-dependent contraction and Rho-kinase activation, leading to sustained vasoconstriction, cellular proliferation, and vascular remodeling. In contrast, endothelial ETB receptors promote nitric oxide and prostacyclin release and facilitate endothelin-1 clearance. In CMD, increased endothelin-1 activity and reduced nitric oxide bioavailability shift this balance toward vasoconstriction, increased microvascular resistance, and impaired myocardial perfusion [60,61].

Selective ETA receptor blockade may reduce pathological vasoconstriction while preserving the beneficial effects of ETB signaling. PRIZE was a phase 2, double-blind, placebo-controlled crossover trial involving 118 patients with microvascular angina. Participants received zibotentan 10 mg daily or placebo for 12 weeks, and the primary endpoint was treadmill exercise duration using the Bruce protocol. The study was completed and published in 2024. Zibotentan did not improve exercise duration compared with placebo or any secondary outcome, while adverse events were more frequent with zibotentan. These findings do not support its current clinical use, although other studies, including EDIT-CAS, continue to evaluate the endothelin pathway in specific coronary vasomotor endotypes [125,126,127,128].

Direct clinical evidence for SGLT2 inhibitors and GLP-1 receptor agonists in ANOCA and INOCA remains limited. In an open-label proof-of-concept study of 33 overweight women with CMD, 12 weeks of liraglutide produced significant weight loss and blood pressure reduction but did not improve coronary flow velocity reserve or angina symptoms. Small phase 2 studies of empagliflozin have reported reductions in inflammatory and endothelial dysfunction markers in women with INOCA, but adequately powered randomized trials assessing myocardial perfusion, symptoms, and clinical outcomes are still needed [129,130,131].

Several device-based and regenerative approaches are also being explored for patients with refractory symptoms. Coronary sinus reducers, CD34 stem cell therapy, and neuromodulation have shown encouraging results in early studies, particularly in patients who remain symptomatic despite optimal medical therapy. However, evidence is still limited, and these interventions cannot yet be recommended for routine clinical practice [132,133]. Most emerging therapies are currently supported by early-phase studies or ongoing clinical trials. Consequently, their place in routine clinical practice remains uncertain until larger randomized outcome studies become available.

Future advances will likely depend on combining precise phenotyping with mechanism-specific therapies. As coronary function testing becomes more widely available and ongoing clinical trials report their results, treatment is expected to move beyond empirical symptom control toward therapies that directly target the biological pathways responsible for coronary microvascular dysfunction. The key principles of this management strategy are summarized in Table 4.

## 6. Summary and Future Perspectives

Despite substantial advances in the understanding of coronary microvascular dysfunction, many patients with ANOCA and INOCA continue to experience delayed diagnosis, repeated testing and persistent symptoms. Wider implementation of standardized coronary function testing and greater clinical awareness of coronary vasomotor disorders are therefore needed.

Future research should focus on more precise disease phenotyping by integrating invasive coronary physiology with quantitative non-invasive imaging, intravascular imaging, circulating biomarkers, and artificial intelligence. These approaches may improve diagnostic accuracy, prognostic stratification and identification of the mechanisms responsible for myocardial ischemia in individual patients.

Further studies are also needed to evaluate therapies targeting endothelial dysfunction, vascular inflammation, oxidative stress, microvascular remodeling, and the endothelin pathway. Adequately powered randomized clinical trials should determine whether mechanism-specific treatment improves not only symptoms and quality of life but also long-term cardiovascular outcomes. Improved access to diagnostic testing and validation of standardized treatment pathways will be essential for translating precision medicine into routine clinical practice.

## 7. Conclusions

Coronary microvascular dysfunction is a major cause of angina and myocardial ischemia in patients without obstructive coronary artery disease. ANOCA and INOCA encompass distinct and frequently overlapping functional endotypes that cannot be identified by coronary angiography alone. Combining quantitative non-invasive imaging with invasive coronary function testing enables more accurate endotype identification and supports mechanism-based treatment. A shift from the exclusion of obstructive disease toward physiological diagnosis is therefore essential for improving the management of these patients.

## Figures and Tables

**Table 1:** Definitions and diagnostic characteristics of ANOCA/INOCA endotypes.

Endotype	Definition	Diagnostic Characteristics
Endothelial dysfunction	Impaired endothelium-dependent regulation of coronary vascular tone resulting in an abnormal vasomotor response before overt vasospasm develops	Abnormal response to low-dose acetylcholine characterized by paradoxical vasoconstriction or inadequate increase in coronary blood flow without fulfilling criteria for vasospastic angina [27]
Structural coronary microvascular dysfunction [CMD]	Structural remodeling of the coronary microcirculation leading to impaired maximal myocardial perfusion	Reduced coronary flow reserve [CFR < 2.5] with increased microvascular resistance [IMR ≥ 25, HMR > 2.5, or reduced MRR], reflecting impaired hyperemic flow [18]
Functional coronary microvascular dysfunction [CMD]	Functional impairment of coronary vasodilatory capacity without substantial structural remodeling of the microcirculation	Reduced CFR [<2.5] despite normal microvascular resistance [IMR < 25], indicating inadequate vasodilatory reserve [18]
Epicardial vasospastic angina	Transient vasoconstriction of an epicardial coronary artery causing myocardial ischemia	Reproduction of typical angina with ischemic ECG changes and ≥90% epicardial coronary constriction during acetylcholine [or ergonovine] provocation testing [11]
Microvascular vasospastic angina	Dynamic vasospasm affecting the coronary microcirculation in the absence of significant epicardial spasm	Typical angina and ischemic ECG changes during acetylcholine testing without ≥90% epicardial Vasoconstriction [28]
Mixed endotype	Coexistence of two or more coronary functional abnormalities [e.g., CMD and vasospasm]	Combination of abnormal coronary flow reserve and/or microvascular resistance together with positive vasoreactivity testing for epicardial or microvascular spasm [19]
Equivocal response/ No demonstrable functional abnormality	Persistent symptoms without objective evidence of CMD or coronary vasospasm during invasive coronary function testing	Normal CFR and microvascular resistance with negative or inconclusive acetylcholine provocation despite symptom reproduction [19,27]

Abbreviations: ANOCA, angina with non-obstructive coronary arteries; INOCA, ischemia with non-obstructive coronary arteries; CMD, coronary microvascular dysfunction; CFR, coronary flow reserve; IMR, index of microcirculatory resistance; HMR, hyperemic microvascular resistance; MRR, microvascular resistance reserve; ECG, electrocardiogram.

**Table 2:** Key pathophysiological mechanisms of coronary microvascular dysfunction.

Mechanism	Primary Abnormality	Main Consequences	Typical Associations
Structural CMD	Arteriolar remodeling, capillary rarefaction, perivascular fibrosis	Reduced coronary flow reserve, increased microvascular resistance, impaired hyperemic perfusion	Hypertension, diabetes mellitus, CKD, obesity, aging, left ventricular hypertrophy, hypertrophic cardiomyopathy
Functional CMD	Impaired vasomotor regulation with preserved microvascular structure	Inadequate vasodilation or paradoxical vasoconstriction during stress	Endothelial dysfunction, vascular smooth muscle dysfunction, sympathetic activation
Endothelial dysfunction, inflammation and oxidative stress	Reduced nitric oxide bioavailability, oxidative stress, chronic vascular inflammation, increased endothelin-1 activity	Impaired endothelium-dependent vasodilation, increased vascular tone, progressive microvascular injury	Cardiovascular risk factors, chronic inflammatory states, CKD, diabetes mellitus
Coronary microvascular spasm	Transient constriction of the coronary microcirculation	Episodic myocardial ischemia despite non-obstructive epicardial arteries	Endothelial dysfunction, enhanced vasoconstrictor activity, Rho-kinase activation
Diffuse non-obstructive atherosclerosis	Mild diffuse epicardial plaque burden with concomitant microvascular dysfunction	Reduced myocardial perfusion and coronary flow reserve despite absence of focal stenosis	Diffuse coronary atherosclerosis, shared cardiovascular risk factors

Abbreviations: CMD, coronary microvascular dysfunction; CKD, chronic kidney disease.

**Table 3:** Comparison of non-invasive and invasive diagnostic modalities for coronary microvascular dysfunction.

Modality	Primary Assessment	Main Advantages	Main limitations	Best Clinical Use
CCTA	Epicardial coronary anatomy	Excellent for excluding obstructive CAD; detects diffuse non-obstructive atherosclerosis	Does not directly assess microvascular function	Initial anatomical evaluation
Stress PET	Myocardial blood flow, CFR	Quantitative MBF and CFR; non-invasive reference standard for CMD	Radiation, high cost, limited availability	Suspected CMD requiring quantitative flow assessment
Stress CMR	Myocardial perfusion, MPR	High spatial resolution; no ionizing radiation; tissue characterization	Limited availability; longer examination time	Assessment of ischemia and myocardial perfusion
Stress echocardiography	Ischemia; CFVR [when Doppler is used]	Widely available; inexpensive; no radiation	Operator-dependent; lower sensitivity for diffuse CMD	First-line evaluation where advanced imaging is unavailable
Invasive coronary function testing [ICFT]	CFR, IMR, endothelial function, vasospasm	Comprehensive physiological assessment; identifies CMD endotypes; guides personalized therapy	Invasive procedure requiring specialized expertise	Persistent ANOCA/INOCA after non-invasive evaluation

Abbreviations: CAD, coronary artery disease; CFR, coronary flow reserve; CFVR, coronary flow velocity reserve; CMD, coronary microvascular dysfunction; CMR, cardiovascular magnetic resonance; CCTA, coronary computed tomography angiography; ICFT, invasive coronary function testing; IMR, index of microcirculatory resistance; MBF, myocardial blood flow; MPR, myocardial perfusion reserve; PET, positron emission tomography.

**Table 4:** Endotype-guided management of ANOCA and INOCA.

Diagnostic Finding	What Does it Suggest?	Clinical Implication
Reduced CFR + high IMR	Structural CMD	Focus on disease-modifying therapy and risk factor control
Reduced CFR + normal IMR	Functional CMD	Improve endothelial function
Positive acetylcholine test with epicardial spasm	Vasospastic angina	Anti-spasm therapy
Positive acetylcholine test without epicardial spasm	Microvascular spasm	Vasodilator-based treatment
Multiple abnormalities	Mixed endotype	Combination therapy and individualized follow-up

Abbreviations: ANOCA, angina with non-obstructive coronary arteries; INOCA, ischemia with non-obstructive coronary arteries; CMD, coronary microvascular dysfunction; CFR, coronary flow reserve; IMR, index of microcirculatory resistance.

## Data Availability

No new data were created or analyzed in this study. Data sharing is not applicable.
